# Exclusive breastfeeding and its effect on growth of Malawian infants: results from a cross-sectional study

**DOI:** 10.1179/2046905514Y.0000000134

**Published:** 2015-02

**Authors:** J Kuchenbecker, I Jordan, A Reinbott, J Herrmann, T Jeremias, G Kennedy, E Muehlhoff, B Mtimuni, M B Krawinkel

**Affiliations:** 1 Institute of Nutritional Sciences, Justus Liebig University Giessen, Germany; 2 Nutrition Division, Economic and Social Department, Food and Agriculture Organization of the United Nations, Rome, Italy; 3 Bioversity International, Rome, Italy (formerly ^2^); 4 Lilongwe University of Agriculture and Natural Resources, Bunda College Campus, Malawi

**Keywords:** Exclusive breastfeeding, Child growth, LAZ, Malawi

## Abstract

**Background:**

For the optimal nutrition of children under 2 years of age, it is considered important that they be exclusively breastfed for the first 6 months before being given complementary food.

**Aims and Objectives:**

A cross-sectional nutritional baseline survey was undertaken in 2011 in the Kasungu and Mzimba Districts of Malawi to assess the nutritional status of children under 2 years of age and its determinants in order to prepare a nutrition education intervention programme. The intention of this study was to assess the nutritional status of infants aged 0–<6 months with regard to food intake.

**Methods:**

Interviews were conducted on randomly selected families with children under 2 years; anthropometric measurements were obtained from mothers and their children. Only infants between 0 and <6 months were selected for analysis (*n*  =  196). An ANCOVA test was performed on age of the infant with mothers’ height and weight as covariates.

**Results:**

Prevalence of stunting (infants’ length-for-age *Z*-score (LAZ) <−2SD) was 39%, wasting (WLZ <−2SD) 2%, and underweight (WAZ <−2SD) 13%. Of the infants under 6 months, 43% were exclusively breastfed. Predominant breastfeeding and mixed breastfeeding were less common (21% and 36%, respectively). The ANCOVA confirmed the association between exclusive breastfeeding and LAZ and WAZ: exclusively breastfed infants had a higher mean (SE) LAZ (−1·13, 0·12) and WAZ (−0·41, 0·13) than infants not being exclusively breastfed (−1·59, 0·11, and −0·97, 0·11, respectively). There was no overall significant association between breastfeeding practice and WLZ.

**Conclusion:**

Exclusive breastfeeding of infants under 6 months is associated with higher mean LAZ and WAZ. Promotion of exclusive breastfeeding in low-income countries is important in preventing growth retardation.

## Introduction

The burden of malnutrition in many developing countries continues to be high and slows the potential for individual, social and economic development. High rates of wasting and stunting in children under 5 years of age reflect the serious challenges faced by many developing countries, including inadequate access to and availability of a healthy, varied diet, improper feeding and caring practices, and poor health and hygiene.^1^–^3^ Chronic malnutrition in infants and children are major problems in Malawi.^4^ In 2010, the Malawi Demographic Health Survey (MDHS) showed that around 47% of children under 5 years of age were classified as stunted (chronic malnourished),^5^ defined as a length-for-age *Z*-score (LAZ) <−2 SD.^6^ Several reviews of nutrition interventions have demonstrated that increased attention needs to be given to complementary feeding interventions targeting children aged 6–23 months. In developing countries, this is the age at which the peak incidence of growth faltering, micronutrient deficiencies and infectious diseases occurs.^8^–^10^ The effects of poor nutrition and poor health and care practices resulting in stunting may also be associated with delayed motor and mental development.^11^,^12^

Since 2008, the Flemish International Cooperation Agency (FICA) funds the Food and Agriculture Organization (FAO) of the United Nations’ project ‘Improving Food Security and Nutrition (IFSN) Policies and Programme Outreach’ in 12 Extension-Planning Areas (EPAs) in Kasungu and Mzimba districts in Malawi. To improve overall food diversity, availability and accessibility, the project focuses on strengthening the agricultural extension system through the establishment of farmer field schools, the distribution of and training on seeds and livestock, and the introduction of improved methods of agricultural production and irrigation systems. The project pays special attention to infant and young child feeding (IYCF) practices to improve food diversity, and has assisted the government in developing teaching materials and training community nutrition facilitators to improve caretakers’ knowledge of IYCF in the programme area after the recommended 6 months of exclusive breastfeeding has elapsed.

For the optimal nutrition of healthy children of this age, it is considered important that they be exclusively breastfed for the first 6 months before being given complementary food. The objective of this study was to assess the nutritional status of infants aged 0–6 months and their breastfeeding (BF) status. This will facilitate understanding the children’s starting point in terms of nutritional status before complementary feeding is commenced.

The study was based on the hypothesis that exclusively breastfed children demonstrate better growth and are less stunted than those who are not exclusively breastfed for the first 6 months of life.

## Methods

### Study area

In 2011, the IFSN project commenced supporting food security and nutrition education in six EPAs in the north of Kasungu and the south of Mzimba District, in central and northern Malawi. Within these EPAs, the project identified 24 sections which were subsequently the sites of the research project. In August/September 2011, a cross-sectional baseline nutrition survey was conducted in the research area before any nutrition education activities were commenced.

### Sampling procedure

The necessary sample size to examine the prevalence of stunting in children aged 0–23 months was estimated. It was estimated that at the time of the survey 8000 children under 2 years were living in the research area. Assuming 47% of stunting, a desired precision of ± 5% and a design effect of 3, the sample size calculated was 1096 children. A two-stage probability sampling strategy was applied. The EPA sections were the primary sampling units. At the first sampling stage, three villages with probability sampling proportional to population size were selected per section using the software ENA for Smart.^13^ At the second sampling stage, 15 households with children under 2 years of age were randomly selected from each village using the software R.^14^ The present analysis includes data on the infants aged 0–<6 months only (*n*  =  196).

### Data collection

Care-givers with children under 2 years of age were interviewed by trained local personnel in their native language, Chichewa or Chitumbuka, using a pre-tested, structured, standardised questionnaire with closed questions. The questionnaire assessed socio-economic variables, food security, the household’s and children’s food intake and breastfeeding pattern, the care-giver’s available time, access to health care facilities, water and sanitation, and the care-giver’s knowledge of food and feeding practices. Data on food and breast-milk intake related to the previous 24 hours.

After the interview, the anthropometric measurements of mothers, children and fathers when possible were recorded. Children were weighed naked while held by their mothers and their length was measured wearing light clothing. Adults’ weights were taken while wearing light indoor clothing and no shoes. Height, length and weight were assessed to the nearest 0·5 cm and 0·1 kg, respectively. All measures were taken twice and the mean was used for analysis. The maximum tolerated difference between the two measurements was 0·7 cm for length and 0·5 kg for weight.^15^ Weight was measured using standardised digital flat scales with mother/child function (Seca 874, capacity 200 kg, SECA, Germany; kg to two decimal points). Children’s recumbent length was obtained using measuring boards (Seca 417, measurement range 10–100 cm, SECA, Germany). Adults’ heights were measured with stadiometers (Seca 213, measuring range 20–205 cm, SECA, Germany).

### Data processing and analysis

Data were double-entered using EpiData 3·1 for MS Windows.^16^ Anthropometric *Z*-scores for the children were generated using the WHO open source software ‘WHO Anthro’.^17^ Only data sets in which the interviewed care-giver was also the biological mother were used for further analysis. If a household had twins, mothers were interviewed about both, but only the data of the child with the registration number one were included in the data analysis. The statistical software SPSS (IBM SPSS version 20.0.0.2.) for MS Windows was used to perform descriptive and explorative data analyses.

A *t*-test was applied to test for differences between means. Differences between male and female infants were tested using the Pearson χ^2^ test for categorical variables, as were differences in group characteristics for exclusively and non-exclusively breastfed infants. An ANCOVA (analysis of co-variance) was performed to determine whether exclusively breastfed infants showed better growth than non-exclusively breastfed ones. Significance was estimated using the F-test at the 5% level. The risk of receiving complementary foods before the age of 6 months was calculated using the Kaplan–Meier life table method for censored data. A quantile regression was applied to estimate the effects of breastfeeding on growth at different length-for-age *Z*-scores by Stata version 10, using the procedure sqreg for simultaneous-quantile regression.

### Anthropometry

The following indicators were used to define the infants’ nutritional status:

- Stunting: length-for-age *Z*-score (LAZ) <−2 SD of the reference population, indicator for long-term nutritional deprivation;
- Wasting: weight-for-length *Z*-score (WLZ) <−2 SD of the reference population, indicator for acute malnutrition;
- Underweight: weight-for-age *Z*-score (WAZ) <−2 SD of the reference population, indicator commonly used for growth monitoring (non-specific malnutrition).

Anthropometric *Z*-scores for WAZ, LAZ and WLZ were calculated on the basis of the WHO growth standards for children <5 year of age.^6^ Mothers’ nutritional status was determined by body mass index [BMI, weight (kg)/height^2^ (m^2^)]. Normal BMI ranged from 18·5 to 25 kg/m^2^. BMI >25 kg/m^2^ indicated overweight and BMI <18·5 kg/m^2^ was taken as underweight.^7^

### Exclusive breastfeeding

Exclusive breastfeeding was defined according to the WHO indicator for IYCF practices, i.e. giving only breast-milk to the infant (directly from the breast or expressed) and nothing else to drink or eat with the exception of vitamin/mineral supplements or medicines within the previous 24 hours.^18^ Non-exclusive breastfeeding was defined as having given breast-milk and other liquids and/or foods within the previous 24 hours. For data analysis, the indicators for breastfeeding practices were summarised into a categorical variable with the value of one for exclusive breastfeeding and two for non-exclusive breastfeeding.

### Episodes of illness

Diarrhoea was determined as perceived by mothers, or as three or more loose or watery stools per day, or blood in the stool. Acute respiratory infection (ARI) was estimated by asking mothers whether their children had been ill with a cough accompanied by short, rapid breathing. The reference period for all illnesses was the 2 weeks before the survey.

### Level of education

Years of schooling were calculated on the following basis: no education, 0 years; some primary education, 4 years; completed primary education, 8 years; some secondary education, 10 years; completed secondary education, 12 years; and more than secondary education, 15 years.

### Ethical approval

The study was granted ethical approval by the Institutional Review Board of the faculty of medicine, Justus Liebig University Giessen, Germany, and by the National Health Sciences Research Committee in Malawi. Participants were not coerced to engage in the study activities and written, informed consent was sought from participants before any data were collected. For illiterate respondents, a thumb print was taken as signature. Confidentiality of the data and the participants’ privacy were respected at all times. The study has been registered with the German Clinical Trials Register in Freiburg, Germany (DRKS00003234).

## Results

In total, 1041 households with children aged <2 years participated in the survey in 2011. Among these, 208 infants were under 6 months. In three cases, the primary care-giver was not the infant’s mother and so they were excluded from the analysis. Of 205 mother–child pairs, nine datasets of anthropometric data for either the infant or the mother were incomplete and were therefore excluded. Multiple imputation was undertaken for the incomplete datasets and included into the models to keep the remaining 205 cases. There was no significant change in the power of the models and the results. Finally, only mother–child pairs with complete datasets were included in the data analyses.

Table 1 illustrates the socio-demographic characteristics of the respondents stratified by breastfeeding practice. The majority of households were of Tumbuka (73%) or Chewa (18%) ethnicity, which is typical of the study region. Households were predominantly male-headed (94%) and household heads had received an average 8 years of schooling. Main source of income was farming (76%). The majority of mothers were married monogamously (85%) and had attended school for an average 6 years. However, 8% never attended school. Maternal BMI ranged from 16·4 kg/m^2^ (minimum) to 29·4 kg/m^2^ (maximum) and most had a normal BMI (88%). Slightly more infants were male (54%).

**Table 1:** Maternal and infant socio-demographic characteristics stratified by breastfeeding practice

		Exclusive	Non-exclusive
Variables	Total	*n* (%)	*n* (%)
Male	105	50 (58·8)	55 (49·5)
Female	91	35 (41·2)	56 (50·5)
Ethnicity:			
Chewa	36	18 (21·2)	18 (16·2)
Tumbuka	143	57 (67·1)	86 (77·5)
Other	17	10 (11·7)	7 (6·3)
Source of income:			
Farming	149	64 (75·3)	85 (76·6)
Other self-employment	23	12 (14·1)	11 (9·9)
Employed	24	9 (10·6)	15 (13·5)
Water and sanitation:*			
Improved water source	158	67 (78·8)	91 (82·0)
Improved sanitation facility	65	34 (40·0)	31 (27·9)
Head of household’s education:			
Don’t know	1	0	1 (0·9)
None	2	1 (1·2)	1 (0·9)
Some primary education	82	25 (29·4)	57 (51·4)
Completed primary education	41	19 (22·4)	22 (19·8)
Some secondary education	34	18 (21·2)	16 (14·4)
Completed secondary/higher education	36	22 (25·9)	13 (12·6)
Mothers’ education:			
None	16	5 (5·9)	11 (9·9)
Some primary education	116	45 (52·9)	71 (64·0)
Completed primary education	30	14 (16·5)	16 (14·4)
Some secondary education	23	15 (17·6)	8 (7·2)
Completed secondary education	11	6 (7·1)	5 (4·5)
Maternal marital status:			
Married, monogamous	166	77 (90·6)	89 (80·2)
Married, polygamous	25	7 (8·2)	18 (16·2)
Single/separated/widowed	5	1 (1·2)	4 (3·6)
Maternal BMI (kg/m^2^):			
<18·5	7	5 (5·9)	2 (1·8)
18·5–24·9	171	72 (84·7)	99 (89·2)
>24·9	18	8 (9·4)	10 (9·0)

* Drinking water from an improved source was defined as: water piped into dwelling, yard or plot, public tap, borehole, protected spring and rainwater. Improved sanitation facility was defined as flush/pour-flush to: piped sewer system, septic tank, pit latrine, unknown place/not known, ventilated improved pit latrine, pit latrine with slab, composting toilet.

**Figure 1:**
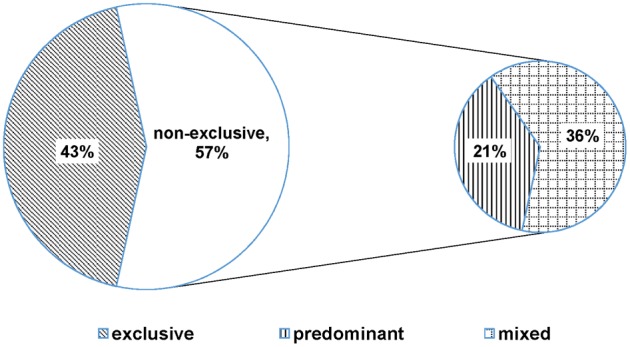
Breastfeeding pattern of study infants at the time of data collection (*n*  =  196)

The prevalence of exclusive breastfeeding of infants 0-< 6 months was 43% (*n*  =  85) and of non-exclusive breastfeeding was 57% (*n*  =  111) (Fig. 1). Exclusive breastfeeding was less common in older infants, ranging from 81% of infants <1 month to 15% of infants aged 5 months. The mean (SD) age of the exclusively breastfed infants was 85·7 days (48·8, 2–178), and of the non-exclusively breastfed infants was 120·1 days (47·68, 16–182, median 130). The non-exclusively breastfed group were further divided into those who received plain water only (36%) and those given various liquids and/or complementary foods (64%).

The liquids and complementary foods given to non-exclusively breastfed infants in the 24 hours before the survey are presented in Table 2. They most commonly received water, thin porridge and other water-based liquids. Complementary foods were mainly from three food groups: grains, roots and tubers; legumes and nuts; and dairy products.

**Table 2:** Frequency of giving liquids and complementary foods in the previous 24 hours to non-exclusively breastfed children

Variable	Total	*n* (%)
Liquids received:	111	
Water		84 (75·7)
Thin porridge		43 (38·7)
Tea/infusion, water-based liquids		36 (32·4)
Milk, yoghurt		6 (5·4)
Juice		5 (4·5)
Infant formula		3 (2·7)
		
Complementary foods received:	71	
Grains, roots & tubers		63 (88·7)
Legumes & nuts		12 (16·9)
Dairy produce		16 (22·5)
Flesh foods		2 (2·8)
Eggs		1 (1·4)
Vitamin A-rich foods		3 (4·2)
Other fruit & vegetables		5 (7·0)

Breastfeeding status did not differ significantly between male and female infants χ^2^ (1)  =  1·67, *P*  =  0·197. There were no significant differences between both groups’ mothers in age, height, weight or BMI.

Table 3 shows the variables tested for association with exclusive and non-exclusive breastfeeding. Mother’s behaviour related to exclusive breastfeeding included skin-to-skin contact within 1 hour of birth, and seeking advice on child feeding from health professionals (χ^2^ (1)  =  4·02, *P*  =  0·045 and χ^2^ (1)  =  8·52, *P*  =  0·004, respectively). Exclusively breastfed infants had significantly fewer episodes of fever and/or diarrhoea during the 2 weeks before the survey (χ^2^ (1)  =  11·26, *P*  =  0·001 and χ^2^ (1)  =  12·13, *P* < 0·001, respectively). Higher education levels of both mothers and heads of households were related to higher rates of exclusive breastfeeding [*t* (193)  =  3·39, *P*  =  0·001 and *t* (168)  =  2·485, *P*  =  0·015, respectively]. There were no significant differences between the groups with regard to early initiation of breastfeeding (<1 hr), gender of head of household, source of income (farming *vs* non-farming), maternal marital status, maternal age (years), maternal BMI (kg/m^2^) and ARI during the previous 2 weeks.

**Table 3:** Variables associated with breastfeeding practices

		Exclusive	Non-exclusive	*P*-value
Variable	Total	*n* (%)	*n* (%)	
Skin-to-skin contact within 1 hr of birth	196	77 (90·6)	89 (80·2)	0·045
Mother received advice on child feeding from health professional	196	74 (87·1)	77 (69·4)	0·004
Had fever within last 2 wks	196	20 (23·5)	52 (46·8)	0·001
Had diarrhoea within last 2 wks	196	9 (10·6)	35 (31·5)	<0·001
Had ARI within last 2 wks	195	42 (49·4)	59 (53·6)	0·558
Breastfeeding initiated within 1 hr 2of birth	196	61 (71·8)	73 (65·8)	0·371
Male-headed household	195	81 (96·4)	102 (91·9)	0·192
Farming is main income	196	65 (76·5)	87 (78·4)	0·751
Mothers’ marital status:	196			
Monogamous		77 (90·6)	89 (80·2)	0·089*
Polygamous		7 (8·2)	18 (16·2)	
Single/separated/widowed		1 (1·2)	4 (3·6)	

	Total	Mean (SD)	Mean (SD)	*P*-value
Average years of schooling:				
Head of household	195	8·30 (3·34)	6·68 (3·19)	0·001
Mother	196	6·07 (3·23)	4·97 (2·92)	0·015
Maternal age, yrs	188	26·7 (5·93)	26·6 (6·44)	0·985
Maternal BMI, kg/m^2^	196	22 (2·22)	22 (2·29)	0·881

* Mann–Whitney *U-*test: differences between exclusive and non-exclusive; ARI, acute respiratory infection.

There were no significant differences between boys and girls in WAZ or WLZ. Only the LAZ score was significantly different [*t* (194)  =  −2·50, *P*  =  0·01]: girls had a higher mean (SD) LAZ [−1·17 (1·11)] than boys [−1·55 (1·02)].

Group differences between exclusive and non-exclusive breastfeeding were analysed after adjustment for the covariates age of child, height and weight of mother, average years of schooling of household head and mother, advice on child feeding from health professionals and skin-to-skin contact within 1 hour of birth. Pairwise comparison demonstrated significantly higher mean values for length and weight of exclusively breastfed infants (Table 4) who on average were 1·08 cm longer and 0·46 kg heavier [F (1, 186)  =  9·82; *P  = * 0·003 and F (1, 186)  =  15·20, *P* < 0·001, respectively). With age as a covariate, there was significant interaction between breastfeeding practice and mean weight. Dis-aggregated into age groups and using the estimated marginal mean of weight (kg) for each month, there were significant differences only for infants aged 2 months [mean  =  5·26, (SE 0·08) *vs* 5·00 (SE 0·10), *P*  =  0·047], 3 months [mean 6·06 (SE 0·09) *vs* mean 5·59 (SE 0·07), *P* < 0·001], 4 months [mean 6·83 (SE 0·11) *vs* mean 6·16 (SE 0·07), *P* < 0·001] and 5 months [mean 7·60 (SE 0·15) *vs* mean 6·74 (SE 0·10), *P*  =  0·001] with higher mean values for exclusive breastfeeding (Table 5).

**Table 4:** ANCOVA* results of breastfeeding practices associated with growth of the child

Variable	Breastfeeding	*n*	Mean	SE	95% CI	*P*-value
Weight (kg)	Exclusive	85	6·03	0·09	5·86–6·20	<0·001
	Non-exclusive	111	5·57	0·07	5·42–5·71	
Length (cm)	Exclusive	85	59·00	0·26	58·48–59·51	0·003
	Non-exclusive	111	57·92	0·22	57·49–58·36	
LAZ	Exclusive	85	−1·13	0·12	−1·37–−0·88	0·007
	Non-exclusive	111	−1·59	0·11	−1·80–−1·38	
WAZ	Exclusive	85	−0·41	0·13	−0·66–−0·17	0·001
	Non-exclusive	111	−0·97	0·11	−1·18–−0·76	
WLZ	Exclusive	85	0·70	0·13	0·44–0·95	0·131
	Non-exclusive	111	0·43	0·11	0·21–0·65	

* Values are differences based on ANCOVA models with covariates at the following values: infant’s age in days, 105·91; mean height of mother, 156·33; mean weight of mother, 53·89; mean years of mothers’ schooling, 5·46; mean years of schooling of household head, 7·38; advice on child feeding from health professional, 1·22; skin-to-skin contact within 1 hr of birth, 1·15.

**Table 5:** ANCOVA* results of breastfeeding practice associated with infant’s, disaggregated into five age groups

Age, mths	Breastfeeding	*n*	Mean, kg	SE	95% CI	*P*-value
0	Exclusive	17	3·69	0·14	3·42–3·97	0·536
	Non-exclusive	4	3·83	0·17	3·49–4·17	
1	Exclusive	10	4·49	0·10	4·29–4·69	0·701
	Non-exclusive	14	4·42	0·13	4·16–4·68	
2	Exclusive	18	5·26	0·08	5·10–5·42	0·047
	Non-exclusive	14	5·00	0·10	4·81–5·19	
3	Exclusive	18	6·06	0·09	5·88–6·23	<0·001
	Non-exclusive	16	5·59	0·07	5·44–5·73	
4	Exclusive	15	6·83	0·11	6·60–7·05	<0·001
	Non-exclusive	24	6·16	0·07	6·02–6·31	
5	Exclusive	7	7·60	0·15	7·30–7·90	<0·001
	Non-exclusive	39	6·74	0·10	6·55–6·93	

* Values are differences based on an ANCOVA model with covariates at the following values: mean height of mother, 156·3; mean weight of mother, 53·9; mean years of mothers’ schooling, 5·46; mean years of schooling of household head, 7·37; advice on child feeding from health professional, 1·22; skin-to-skin contact within 1 hr of birth, 1·15.

ANCOVA confirmed the significant influence of breastfeeding practices on LAZ and WAZ [F (1,185) 7·48, *P*  =  0·007, F (1,186) 10·77, *P*  =  0·001, respectively): exclusively breastfed infants had higher mean (SE) LAZ [−1·13 (0·12)] and WAZ [−0·41 (0·13)] than those who were not being exclusively breastfed [−1·59 (0·11), −0·97 (0·11), respectively]. There were no significant differences in WLZ scores between the groups. However, the interaction between breastfeeding practice and the infant’s age was significant for WLZ, leading to a second detailed analysis disaggregated into age groups. This analysis confirmed the significant differences between exclusive and non-exclusive breastfeeding for infants aged 4 and 5 months: those who were exclusively breastfed had higher mean (SE) WLZ [0·81 (0·17) *vs* 0·28 (0·11), *P*  =  0·012 and 0·92 (0·23) *vs* 0·14 (0·15), *P*  =  0·005, respectively] (Table 6).

**Table 6:** ANCOVA* results of breastfeeding practice associated with infant’s WLZ, disaggregated into five age groups

Age, mths	Breastfeeding	*n*	Mean	SE	95% CI	*P*-value
0	Exclusive	17	0·37	0·21	−0·05–0·78	0·150
	Non-exclusive	4	0·85	0·26	0·34–1·37	
1	Exclusive	10	0·48	0·16	0·17–0·79	0·373
	Non-exclusive	14	0·71	0·20	0·32–1·10	
2	Exclusive	18	0·59	0·12	0·34–0·83	0·917
	Non-exclusive	14	0·57	0·15	0·28–0·86	
3	Exclusive	18	0·70	0·13	0·44–0·96	0·119
	Non-exclusive	16	0·42	0·11	0·20–0·64	
4	Exclusive	15	0·81	0·17	0·47–1·15	0·012
	Non-exclusive	24	0·28	0·11	0·06–0·50	
5	Exclusive	7	0·92	0·23	0·46–1·37	0·005
	Non-exclusive	39	0·14	0·15	−0·15–0·42	

* Covariates appearing in the model are evaluated at the following values: mean height of mother, 156·33; mean weight of mother, 53·89; mean years of mother’s schooling, 5·46; mean years of schooling of household head, 7·37; advice on child feeding from health professional, 1·22; skin-to-skin contact within 1 hr of birth, 1·15.

**Figure 2:**
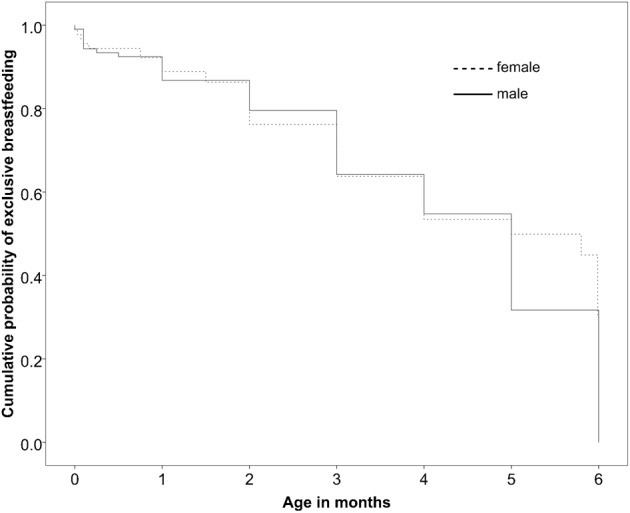
Cumulative probability of exclusive breastfeeding (survival function)

Kaplan–Meier analysis demonstrated that, on average, complementary food was introduced to boys at 4·03 months (SE 0·213) and to girls at 4·2 months (SE 0·243) (Fig. 2). However, the difference was not significant.

**Figure 3:**
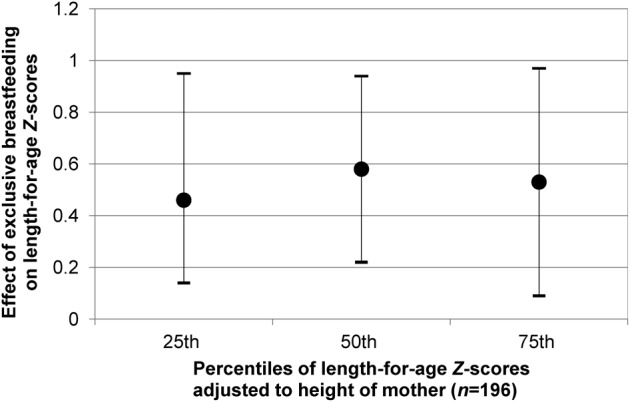
Effect of exclusive breastfeeding on length-for-age *Z*-score (rhombi mark the mean effect, the crosses above and below indicate the 95% CI; model covariates: infant’s gender, mother’s education; bootstraps 2000; pseudo R^2^ of 25th–75th percentiles: 0·06, 0·07 and 0·08, respectively

Quantile regression estimated an effect of breastfeeding on growth at the 25th, 50th and 75th percentiles with length-for-age *Z*-scores of 0·46 (95% CI 0·14–0·95), 0·58 (95% CI 0·22–0·94) and 0·53 (95% CI 0·09–0·97), respectively (all *P* < 0·05) (Fig. 3). This significant difference in LAZ of around 0·5 between exclusively and non-exclusively breastfed infants was observed regardless of whether they had a low, medium or high LAZ.

## Discussion

Infants under 6 months of age who were exclusively breastfed were longer, heavier and less likely to be stunted than non-exclusively breastfed infants. This effect was equally high at the different LAZ percentiles tested in the quantile regression.

Introduction of any other liquid or food apart from breast-milk, especially before the age of 4 months, is associated with increased risk of gastro-intestinal disease, which may result in growth retardation, micronutrient deficiencies and vulnerability towards various infectious diseases within the first 2 years of life.^2^,^19^ Although higher rates of diarrhoea and fever could lead to stunted growth, no significant association between growth retardation in infants with diarrhoea and/or fever and their breastfeeding status could be detected. Prevalence of diarrhoea and/or fever was assessed by asking the mother about episodes in the 2 weeks before the data were collected. More detail about the number of times these illnesses occurred and their severity and duration might be required to significantly link them with growth retardation.

The rates of diarrhoea and fever were found to be higher among non-exclusively breastfed infants. As 6 months of exclusive breastfeeding is often associated with lower rates of respiratory and gastro-intestinal tract infections, the findings here confirm those of other studies.^19^–^22^ There was no significant difference between the groups in access to safe drinking water (exclusively breastfed group 79%, non-exclusively breastfed 82%). Therefore, differences in the incidence of diarrhoea might be related to hygiene and food safety practices rather than to the source of drinking water.

Liquids and/or complementary foods before the recommended age of 6 months were given to 57% of the 208 infants surveyed.^23^ Compared with national data, the data on exclusive breastfeeding deviate from the 2010 MDHS findings in which 71% of infants aged 0–5 months were exclusively breastfed, almost 30% more than in this study.^5^ A much lower rate, only 4% of infants exclusively breastfed for 6 months, was found in another Malawi study in which 65% of the children received, for example, a herbal infusion, water or porridge in the first month of life.^24^ The differences might be explained by different methods of assessing exclusive breastfeeding. In this study exclusive breastfeeding was assessed using the 24-hour recall method recommended by WHO.^18^ Thus, recall bias cannot be excluded. In addition, urban *vs* rural and seasonal differences might play a role. Finally, as the official recommendation is ‘6 months exclusive breastfeeding’, there might have been some over-reporting following national campaigns when the MDHS was conducted.^25^

Early initiation of breastfeeding is important for both mother and child. The exclusively breastfed infants experienced skin-to-skin contact within an hour of birth, significantly more often than in those who were non-exclusively breastfed. Less than 75% of infants in the exclusively breastfed group and around 65% in the non-exclusively breastfed group were put to the breast within 1 hour of birth. These findings for early initiation of breastfeeding differ from the MDHS which reported approximately that 95% of all infants were put to the breast within an hour of birth.^5^ Again, the difference might be explained by regional variance (urban *vs* rural) and over-reporting in the national survey. Early initiation of breastfeeding is considered to be important as it has been found in Kenya to be associated with longer duration and higher rates of exclusive breastfeeding in general.^26^

Breastfeeding in the first days of life provides the newborn with colostrum which is rich in nutrients and antibodies which are important for the development of the intestinal microbiota and the immune system.^23^,^27^,^28^ Colostrum is secreted only within the first 2–3 days after delivery. Almost all children in this study (97%) were breastfed within a day of delivery, and they therefore received colostrum.

As observed in other studies, giving traditional liquids and early complementary feeding is common in Malawi.^24^,^29^ In particular, giving water to infants is widespread and considered a necessity for optimal growth and health in Malawi as well as in other eastern sub-Saharan countries such as Tanzania and Mozambique.^30^,^31^ The negative effect of complementary feeding before the age of 6 months and growth retardation has also been documented in various other studies.^24^,^32^,^33^

A prospective cohort study in southern Malawi in 1996 showed that 30% of the infants observed received complementary foods within the first month of life.^34^ This is similar to results of an earlier prospective cohort study in 1993–1994, also in Southern Malawi, which reported that 40% of children aged 2 months were given complementary foods.^35^ The national MDHS for 2010 reported that 10% of infants up to 3 months of age received complementary feeds, indicating a behaviour change. However, the proportion in infants aged 4–5 months dramatically increased to over 45%.^5^ In our study, 50% of male and female infants aged 4 and 5 months had received complementary food, thus, slightly higher than the national level. Much remains to be done to inform families of the health risks associated with the early introduction of complementary feeding and to support mothers practicing exclusive breastfeeding for 6 months.

Almost all mixed-fed infants (89%) in this study received complementary food such as grains, roots or tubers, which are used to prepare porridge such as ‘phala’. The porridge is usually a watery, maize-based porridge of very limited nutritional value.^5^,^24^,^34^,^35^

In Malawi, an important factor in child nutrition, health and growth is the high prevalence (13%) of HIV-infected women of reproductive age (15–49 years).^5^ Global guidelines for HIV-infected mothers recommend exclusive breastfeeding when breast-milk replacement is not acceptable, feasible, affordable, safe or sustainable. Several studies have demonstrated that postnatal mother-to-child-transmission through breastfeeding is less likely in infants who are exclusively or predominantly breastfed than in those given mixed feeds in the first 6 months of life.^36^–^38^ Therefore, exclusive breastfeeding for the first 6 months needs to be promoted and encouraged to prevent mother-to-child-transmission of HIV. In this regard, it is critical that the majority of Malawian mothers breastfeed their infants for longer with around 80% of children under 2 years still being breastfed.^5^ This practice requires even more information and counselling since children of HIV-infected mothers are at risk of infection if breast problems are not treated promptly.^39^ Therefore, WHO recommends the provision of specific guidance and support to HIV-infected and breastfeeding mothers to avoid harmful nutritional and psychological consequences and to maintain breast health.^40^

This study’s data show a positive association between advice from health workers and exclusive breastfeeding. This might be the result of increased governmental promotion of exclusive breastfeeding in the national policy for IYCF in Malawi.^25^ In a study nearly 20 years ago in southern Malawi, advice on child feeding by health professionals (including breastfeeding) was negatively associated with duration of exclusive breastfeeding.^34^ This seems to have changed, at least in urban areas where women delivering with attendance by a trained health professional are more likely to practice exclusive breastfeeding, as was shown recently in Kenya.^41^ The potential of pro-breastfeeding policies to improve infants’ nutritional status is remarkable, given that infant feeding practices are influenced by many traditional and cultural factors, apart from knowledge and the difficulties of breastfeeding which mothers experience.^26^,^42^–^45^

This study has some limitations. The validity of defining exclusive or non-exclusive breastfeeding only according to 24-hour recall might be questioned;^46^ according to WHO, infants can be classified as exclusively breastfed even when they have received early supplementary feeding and traditional fluids.^18^ Health data, such as on fever and diarrhoea, were reported by mothers without any proof of signs and symptoms; duration of ill-health was not assessed and only a period of 2 weeks before the survey was covered.

However, this study does confirm the importance of exclusively breastfeeding infants for the first 6 months, especially in low-income countries such as Malawi. Exclusive breastfeeding is associated with better growth of infants under 6 months and should be further promoted as a factor in reducing stunting. The study identified success in increasing exclusive breastfeeding rates as well as identifying further requirements for health and nutrition education interventions within the national public health nutrition programmes.
